# Basin stability in delayed dynamics

**DOI:** 10.1038/srep21449

**Published:** 2016-02-24

**Authors:** Siyang Leng, Wei Lin, Jürgen Kurths

**Affiliations:** 1 School of Mathematical Sciences, LNSM and Centre for Computational Systems Biology, Fudan University, Shanghai 200433, China; 2 Potsdam Institute for Climate Impact Research (PIK), Potsdam 14473, Germany; 3 Department of Physics, Humboldt University, Berlin 12489, Germany; 4 Institute for Complex Systems and Mathematical Biology, University of Aberdeen, Aberdeen AB24 3UE, United Kingdom

## Abstract

Basin stability (BS) is a universal concept for complex systems studies, which focuses on the volume of the basin of attraction instead of the traditional linearization-based approach. It has a lot of applications in real-world systems especially in dynamical systems with a phenomenon of multi-stability, which is even more ubiquitous in delayed dynamics such as the firing neurons, the climatological processes, and the power grids. Due to the infinite dimensional property of the space for the initial values, how to properly define the basin’s volume for delayed dynamics remains a fundamental problem. We propose here a technique which projects the infinite dimensional initial state space to a finite-dimensional Euclidean space by expanding the initial function along with different orthogonal or nonorthogonal basis. A generalized concept of basin’s volume in delayed dynamics and a highly practicable calculating algorithm with a cross-validation procedure are provided to numerically estimate the basin of attraction in delayed dynamics. We show potential applicabilities of this approach by applying it to study several representative systems of biological or/and physical significance, including the delayed Hopfield neuronal model with multistability and delayed complex networks with synchronization dynamics.

The linear stability theory plays a central role in complex systems science, which describes the long-term behavior of system’s steady states, such as equilibriums and periodic orbits, after a small perturbation. Such a linearization-based approach has many applications both theoretically and practically by determining whether the steady state is stable or not (in terms of the sign of the Lyapunov exponents)^1^. However, due to the localness of this approach, it is only valid when the perturbation is limited to a small range. Since non-small perturbations are omnipresent in real-world systems, a global method for complementing the linear-stability paradigm needs to be developed. Although the classical Lyapunov function method with the Invariance Principle could be a candidate^2^,^3^,^4^,^5^,^6^, this method, which usually requires subtle construction techniques and highly depends on the accurate form of the systems investigated, has some theoretical significance but lacks of extensively practical usefulness. Fortunately, the basin stability (BS), proposed recently by Menck *et al.*, becomes such a global method, which uses the basin of attraction instead to fully assess the steady state’s stability^7^. This method focuses on the basin’s volume which serves as a measure of the likelihood of return to a steady state after being subjected to any random perturbation^8^ and thus explains to what extent a steady state is stable in a probability sense.

The BS approach has become a powerful tool for complex systems studies and has already been successfully applied to many fields of science^7^,^9^,^10^,^11^, especially in those systems with multi-stability. As a matter of fact, the phenomenon of multi-stability is even more ubiquitous in delayed dynamical systems^9^,^12^,^13^,^14^. Moreover, due to a certain amount of time duration for the signal transmission, such as the finite switching speed and the memory effect, time delay exists in almost every real system^15^,^16^,^17^,^18^. Therefore, it is of great significance to consider the BS in delayed dynamics. To this end, we need to define the basin’s volume in a time-delayed sense, while the space for the initial values of a time-delayed differential equation (DDE) is a function space which has dimension of infinity^19^. Although by measure theory, it is possible to construct analytical measures, such as the Hausdorff measure, to assess the basin’s ‘volume’^20^, such measures are less geometric intuitive or numerical computable as basin’s area or volume in the traditional BS theory of ordinary differential equations.

To overcome these difficulties, we articulate in the following the concept of the BS by a technique which projects the infinite dimensional initial state space of the DDE to a finite dimensional Euclidean space. A function of the initial value is related to its coefficients under the expanding along with an orthogonal or a nonorthogonal basis. Through this, a generalized notion of basin’s volume is theoretically described and a practical calculating algorithm with a procedure of cross-validation is introduced. We then apply the developed approach to study a multi-stable Hopfield neuronal model with time delays. Some further applications, including the investigations of the synchronization of delayed complex networks and multi-stability of equilibriums and periodic orbits in time-delayed oscillators, show the broad potential of applications of this developed approach.

## Results

Consider a system described by the delayed differential equation


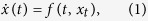


where *x* is an *s*-dimensional state variable, 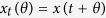 for 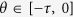, and *τ* is a time delay. The set, denoted by 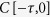, for the initial values of system (1) is a space consisting of continuous functions. We use 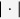 to represent the Euclidean norm in an *n*-dimensional. Now, we state the definition of basin of attraction for a stable steady state, which could be either an equilibrium or a periodic orbit.

### Basin of attraction for delayed dynamics

Suppose 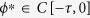 is an isolated asymptotically stable equilibrium or periodic orbit of system (1). If there exists a region 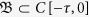 for initial values such that for any 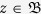, the solution of the system starting from *z*, denoted by 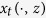, uniformly converges to ϕ^*^ as *t* → +∞, i.e. 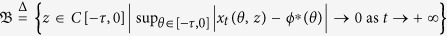, then 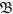 is called the basin of attraction (or basin) with respect to the stable steady state ϕ^*^.

Because the function space for the initial values is taken into account, the basin of attraction of system (1) is of infinite dimension. A direct computation for measuring this kind of BS is impossible, so that some approximate algorithm should be adopted. Notice that, for any 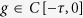 and a given standard orthogonal basis 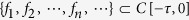, there exists a unique sequence of numbers 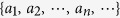 such that


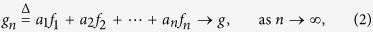


where the limit is interpreted in a sense of the square integral on 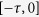 and 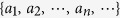 are the Fourier coefficients with 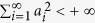
21. Refer to the section of Method for specific bases that we use in the paper. We thus construct a set through 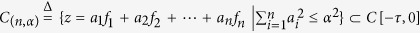, where *α* is a sufficiently large positive constant and 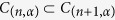. Due to the one-to-one property between *C*_(*n*,*α*)_ and the bounded set 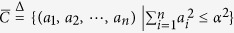 in 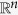, we use the conventional Lebesgue measure *m* to count the volume of *C*_(*n*,*α*)_ as 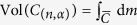. We call the volume of the geometry 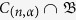 the *n*th-order generalized basin’s volume with respect to the given standard orthogonal basis (also called basin’s volume if not confused). We further normalize this volume through


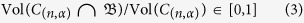


and denote this normalized value by 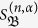. 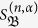 is then used to represent the *n*th-order approximate BS of the basin 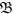 for the delayed dynamics (1).

For the sake of practical use, we introduce how to numerically compute the generalized BS defined above. We integrate system (1) for *T* points drawn uniformly at random from 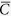, which correspond to *T* initial functions [a point 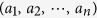 is mapped to the function *g*_*n*_ defined in (2)], and then count the number *M* of the initial functions that finally arrive at the stable steady state ϕ^*^. 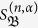 can then be estimated as *M*/*T* when *T* is sufficiently large. In Supplementary Materials, we analytically show how to choose a proper value of *T* for getting a credible estimation on the *n*th-order approximate BS.

Moreover, the generalized BS, as defined in (3), depends on both *n* and the ranges of the coefficients *a*_*i*_. Analytical proof of the BS convergence with increasing either *n* or the range is hard at present. However, numerical validation of this convergence could be adopted in applications, which is shown in the following examples as well as the examples in Supplementary Materials. In fact, when the fluctuations of 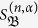, persistently for larger *n*’s or wider ranges, are below an allowable bias specified *a priori*, we take the smallest value of such *n*’s as the number of the basis functions that are successively taken from the standard orthogonal basis, and take the smallest value of such boundaries for the coefficient *a*_*i*_ used in *C*_(*n*,*α*)_.

Clearly, the above procedure for defining and computing the generalized BS for delayed dynamics could be performed not only with the orthogonal bases, such as the trigonometric and the Legendre bases (refer to the section of Method), but also with the nonorthogonal bases, including the Bernstein basis^22^ (refer to Supplementary Materials). Then, in order to avoid losing the essential functions for the initial values in computation, we determine *n* and the ranges for the coefficients through a cross-validation procedure in applications, that is, they take values such that the aforementioned convergence requirements on the approximate BS are satisfied simultaneously for three different bases. Interestingly, as shown in the following examples, although the exact values of the BS for different bases are not uniform, they display almost the same changing tendency with the variation of some parameters such as the time delay.

#### Delayed Hopfield neuronal model

**Figure 1:**
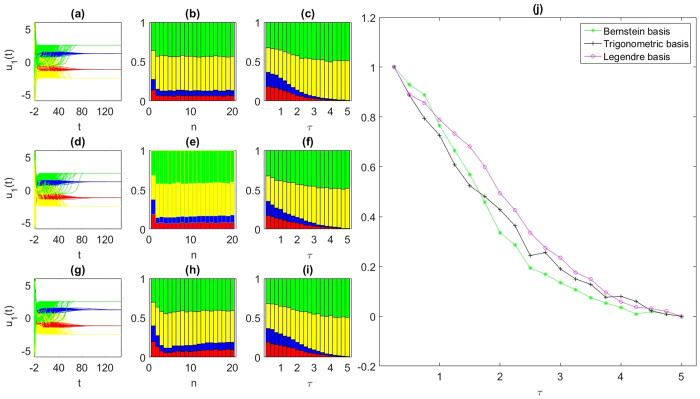
Basin stability of the delayed Hopfield neuronal model with respect to three different bases. Upper, middle and lower graphs correspond to the Bernstein, the trigonometric and the Legendre basis, respectively. (**a**,**d**,**g**) display the integral trajectories of *u*_1_ in the model (4) starting from the initial functions selected from different function spaces for *τ* = 2. All trajectories converge to the four different equilibriums *E*_1,2,3,4_, which are denoted, respectively, by colors of red, blue, green, and yellow. (**b**,**e**,**h**) show that for all the bases, the fluctuations of 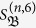 tend to be slight when *n* is sufficiently large. (**c**,**f**,**i**) show analogous landscapes where the proportions of 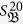 for the equilibriums *E*_1,2_ decrease to almost zero with an increase of *τ*, while the proportions for the other two equilibriums *E*_3,4_ become dominantly equal. (**j**) For the equilibrium *E*_1_, the normalized variations of 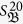 with *τ* for all the bases approach a high consensus.

We first apply the generalized BS approach to the delayed neuronal model. Multi-stability exists widely in neural networks especially when applied to associative memory^23^,^24^. Several sample patterns (represented as equilibriums) are stored in the weights of networks, and associative memory is used to recover from an incomplete or damaged version to its complete pattern with a help of network dynamics. The number of equilibriums represents the capacity of memorial storage, and the basin’s volume is the error correcting capability that determines which memory is of the highest possibility to be remembered. By our approach, we can estimate the volume of several patterns relatively and discover its relationship with the neuron’s parameters. We demonstrate it by considering a simple two-dimensional delayed Hopfield neuronal model^25^, described by


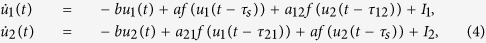


where *u*_*i*_(*t*) is the state of neuron *i* at time *t*. Parameters *a*, *b*, *a*_12_, *a*_21_, *I*_1_ and *I*_2_ are constants with *a* > 0, *b* > 0, and *a*_*ij*_ ≥ 0 for *i*, *j* = 1, 2 and *i* ≠ *j*. 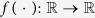 is a nonlinear neuronal activation function. Here, the parameters are set as *a* = 2, *a*_12_ = *a*_21_ = 0.55, *b* = 1, *I*_1_ = *I*_2_ = 0, and *f*(*x*) = tanh(*x*). Time delays in the equations represent the transmission times between different neurons and here we let *τ*_*s*_ = *τ*_12_ = *τ*_21_ = *τ*. By linear stability theory, the model (4) with 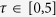 has four asymptotically stable equilibrium points, i.e., 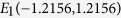, 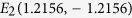, 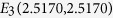, and 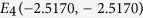, as shown in Fig. 1(a,d,g). For *τ* > 0, these equilibriums correspond to four constant function vectors on the interval [−*τ*,  0], respectively. In the meanwhile, looking into the BS reveals us more interesting detail information about these equilibriums besides whether they are stable or not.

First, in order to find the appropriate number of basis functions in calculating the *n*th-order approximate BS for the four equilibrium points, we use the algorithms with a cross-validation procedure introduced above. In particular, take in account the Bernstein, the trigonometric and the Legendre bases simultaneously, set *α* = 6, and denote by 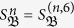 for simplicity. As shown in Fig. 1(b,e,h), the fluctuations of 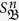 for every basis tend to be stable when *n* is sufficiently large, and thus for the model (4), we take *n* = 20 as the number of the basis functions that are successively selected from the given bases. Since 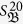 represents the possibility of a pattern that might be remembered, investigating the variation of its value with some parameters becomes an important issue. As shown in Fig. 1(c,f,i), values of 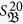 for the two equilibriums *E*_1,2_ are getting smaller and smaller when the time delay *τ* increases (showing a less probability of being remembered), and finally the two other patterns, *E*_3,4_, become dominant with an almost equal proportion. For all the three bases, Fig. 1(j) depicts the normalized curves of the variation of 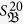 with *τ* for the attractor *E*_1_. As mentioned above, despite the fact that values of 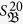 for different bases are not exactly uniform, the variations of these values with the time delay approach a high consensus.

While human brains have a large amount of neurons, the capacity of memorial storage is even enormous. As shown by the example, the BS provides us a potential method to fully discover the multi-attractors and their basins. And comparing to the linearization approach, the BS proves itself a practical tool which provides a global view of the system in a probability sense.

#### Application to complex networks with time-delayed couplings

To show the applicability of this approach even to delayed complex networks, we apply it to study the synchronization phenomenon here. Synchronization is a fundamental nonlinear behavior^26^, which can be observed in many systems such as the firing neurons^13^, the human cardiorespiratory system^27^, the singing crickets and the power grids^9^. The original BS theory shows us to what extent a system will maintain synchronous behavior when perturbations occur and the relationship between networks’ topology and the basin’s volume. An amazing discovery is that the BS of a network shows a quite different trend versus the Watts-Strogatz network’s parameter *p*, compared to the traditional indicator, synchronizability *R*, which is always used to measure the network’s capability of reaching synchronization^7^.

We thus investigate the generalized BS of the complex networks where we take into account the time delay, which may affect the system’s stability of synchronization or even change the synchronization manifold^28^,^29^. We consider an undirected complex network with *N* delay-coupled identical nodes, which is represented as follows^30^:


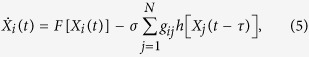


where 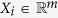 and 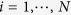. The dynamics of the individual units is governed by the function *F*, *σ* denotes the overall coupling strength constant, and the network’s Laplacian *G* = *g*_*ij*_ is an *N* × *N* matrix which determines the topology of the network. *h* is a coupling function (we choose it as an identity map here) and *τ* is the coupling delay. Under the requirement of 
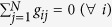
, system (5)’s synchronization manifold is 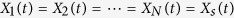. By considering the largest transverse Lyapunov exponent of the master stability function (MSF)^31^, for a large amount of networks we can obtain a range of the normalized coupling parameter *ε* which depends on coupling strength *σ* and coupling matrix *G* (see Methods). In this range, system (5) can be stably synchronized. In the following discussion, the Watts-Strogatz networks consisting of the paradigmatic Rössler oscillators are taken into account^32^. A single Rössler oscillator with given parameters is governed by


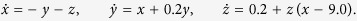


Here, the *x* → *x* component coupling is used to build the network^30^. According to the MSF method, the stable synchronous state exists only in the range of the normalized coupling parameter 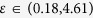 in the absence of the coupling delay. As *τ* increases to 0.24, a second stable regime *ε* > 7.38 emerges and when *τ* reaches 0.4, again only one stable regime exists with 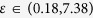
33. It is easy to calculate the range of *σ* according to *ε* and different network topology *G* (see Methods). In the following we concentrate on the regime *τ* = 0.4. The rewiring probability *p* in the Watts-Strogatz network (*p* = 0 for regular lattices and *p* = 1 for random graphs) is varied to see the relationship between network topology and its synchronization.

**Figure 2:**
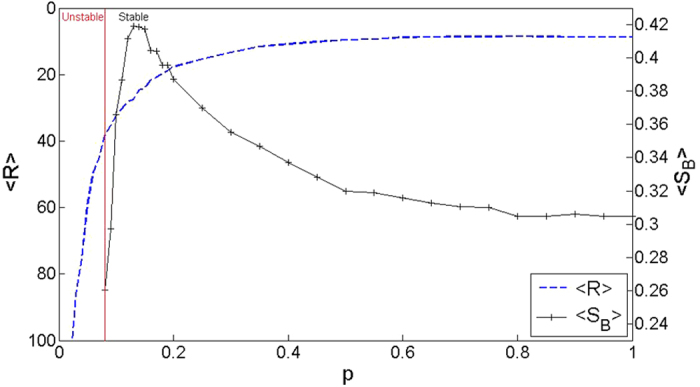
Basin stability of the Watts-Strogatz networks consisting of the delay-coupled paradigmatic Rössler oscillators. Results were obtained for *N* = 100 oscillators, each having on average 6 neighbours. The red vertical line separates the unstable and stable regimes.

From the traditional viewpoint, network’s synchronizability *R*, defined as *γ*_max_/*γ*_min_, the ratio of matrix *G*’s maximum and minimum non-zero eigenvalues (the smaller *R* is, the more synchronizable the network is), always indicates an improvement with the increasing of *p* (from unstable regime to stable regime), see the blue dashed curve plotted in Fig. 2. However, akin to the results for the non-delayed networks^7^, the mean basin stability (see Methods for details) shoots up when *p* is small and reaches a peak. After that, just opposite to the variation of *R* with *p*, an exponential decline of the mean basin stability can be observed, which means the network becomes more synchronizable but the basin of the synchronization manifold is otherwise decreasing. Clearly, this application of the generalized BS approach on delayed networks also complements the linear synchronization studies.

Moreover, this generalized BS approach could also be applied to systems with periodic orbits or even chaotic attractors, which would reveal more subtle nonlinear properties (refer to Supplementary Materials for examples including the delayed Van der Pol-Duffing oscillator model and the delay coupled Stuart-Landau system). All these examples show that the generalized BS approach for delayed dynamics could provide a relatively comprehensive knowledge of the basin of the steady state and shows whether a perturbation is permissible, which is quite essential in engineering.

## Conclusions

The linear stability theory provides a local method to determine whether a state is stable or not. However, limited by the infinite dimension of the initial state space, it is still difficult to have a comprehensive understanding of attractors in delayed dynamics. Although the method of Lyapunov-Krasovskii functional could be used to analyze the multi-stability and basins of multi-attractors, this method still produces a sufficient condition, lacking a capacity of describing the exact shape of an attractor’s basin^4^,^19^, and also this method in essence still restricts the discussion in the finite-dimensional space, describing the condition on initial value only at a time of zero^34^,^35^. To overcome these difficulties, we propose here an approximate approach of applying basin stability to delayed dynamics. The BS approach turns our eyes from a traditional local linearized view of stability to a global inspection. And our method extends its application and converts an infinite dimensional problem into a highly practicable algorithm especially when time delay plays an important role in real-world systems. The generalized BS approach even provides an opportunity to fully investigate more nonlinear properties of attractors in delayed dynamics such as the bifurcation phenomena. Furthermore, an analytical proof on the convergence of our proposed approach for computing the generalized BS is also of great significance, which becomes a topic of our present and future works.

## Methods

### Orthogonal bases

We take, respectively, the orthogonal basis consisting of trigonometric functions on [−*π*, *π*] as follows:


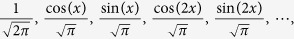


and the Legendre basis on [−1, 1], which is also an orthogonal basis, as follows:


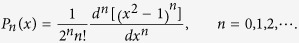


In the framework of functional analysis, a series, which is generated by expanding a function along with a given standard orthogonal basis, could be regarded as a Fourier series^21^. Moreover, in order to realize the cross-validation procedure formalized in the section of Result, we also use the nonorthogonal Bernstein basis, which is introduced in Supplementary Materials.

Remember that different basis leads to different 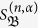. So we should make clear which basis is used if we need to focus on the exact value of 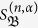. As shown in the above examples and examples in Supplementary Materials, almost the same results on the changing tendency can be obtained while considering the variation of the generalized basin’s volume with some parameters.

### Master stability function

The stability of the synchronization manifold is determined by the eigenvalues of the system’s Laplacian *G* and the following variational equation (i.e., MSF)^31^


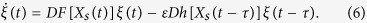


Here, *ξ* is an *m*-dimensional variation transverse to the synchronization manifold. The normalized coupling parameter *ε* = *σγ*_*k*_, in which *γ*_*k*_ denotes the eigenvalues of *G* for 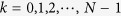. Thus, the stability of the synchronized state of a given network topology *G* is guaranteed by the negative sign of the largest transverse Lyapunov exponent of Eq. (6) 30.

### Estimation of mean basin stability

Notice that the stable synchronous state requires *σ* to take its value in a specific range. Here, we estimate the basin stability for several different equally spaced values in this range and average to obtain a mean 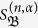. Additionally, several networks are produced for one probability *p* to acquire the average value of 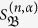.

## Additional Information

**How to cite this article**: Leng, S. *et al.* Basin stability in delayed dynamics. *Sci. Rep.* **6**, 21449; doi: 10.1038/srep21449 (2016).

## Supplementary Material

Supplementary Information

## References

[b1] Pikovsky A., Rosenblum M. & Kurths J. Synchronization: A Universal Concept in Nonlinear Sciences, vol. 12 (Cambridge University Press, Cambridge, 2003).

[b2] LaSalle J. P. The Stability of Dynamical Systems (SIAM, Philadelphia, PA, 1976).

[b3] Rodrigues H. M., Alberto L. F. C. & Bretas N. G. Uniform invariance principle and synchronization. Robustness with respect to parameter variation. J. Differential Equations 169, 228–254 (2001).

[b4] Rabelo M. & Alberto L. F. C. An extension of the invariance principle for a class of differential equations with finite delay. Adv. Difference Equations 2010, 496936 (2010).

[b5] Ge T., Lin W. & Feng J. Invariance principles allowing of non-Lyapunov functions for estimating attractor of discrete dynamical systems. IEEE Transactions on Automatic Control 57, 500–505 (2012).

[b6] Zhong W., Lin W. & Ruan J. The generalized invariance principle for dynamic equations on time scales. Appl. Math. Comput. 184, 557–565 (2007).

[b7] Menck P. J., Heitzig J., Marwan N. & Kurths J. How basin stability complements the linear-stability paradigm. Nature Physics 9, 89–92 (2013).

[b8] Wiley D. A., Strogatz S. H. & Girvan M. The size of the sync basin. Chaos: An Interdisciplinary Journal of Nonlinear Science 16, 015103 (2006). 10.1063/1.2165594 16599769

[b9] Schultz P., Heitzig J. & Kurths J. Detours around basin stability in power networks. New Journal of Physics 16, 125001 (2014).

[b10] Ji P. & Kurths J. Basin stability of the Kuramoto-like model in small networks. The European Physical Journal Special Topics 12, 2483–2491 (2014).

[b11] Menck P. J. & Kurths J. Topological identification of weak points in power grids. In Nonlinear Dynamics of Electronic Systems, Proceedings of NDES 2012, 1–4 (VDE, 2012).

[b12] Lucarini V., Pascale S., Boschi R., Kirk E. & Iro N. Habitability and multistability in earth-like planets. Astronomische Nachrichten 334, 576–588 (2013).

[b13] Shayer L. P. & Campbell S. A. Stability, bifurcation, and multistability in a system of two coupled neurons with multiple time delays. SIAM Journal on Applied Mathematics 61, 673–700 (2000).

[b14] Wang H., Hu H. & Wang Z. Global dynamics of a Duffing oscillator with delayed displacement feedback. International Journal of Bifurcation and Chaos 14, 2753–2775 (2004).

[b15] Wang Q., Duan Z., Perc M. & Chen G. Synchronization transitions on small-world neuronal networks: Effects of information transmission delay and rewiring probability. EPL-Europhysics Letters 83, 50008 (2008).

[b16] Wang Q., Perc M., Duan Z. & Chen G. Delay-induced multiple stochastic resonances on scale-free neuronal networks. Chaos: An Interdisciplinary Journal of Nonlinear Science 19, 023112 (2009). 10.1063/1.3133126 19566247

[b17] Wang Q., Perc M., Duan Z. & Chen G. Synchronization transitions on scale-free neuronal networks due to finite information transmission delays. Physical Review E 80, 026206 (2009). 10.1103/PhysRevE.80.026206 19792230

[b18] Lakshmanan M. & Senthilkumar D. V. Dynamics of Nonlinear Time-Delay Systems (Springer, Berlin, 2011).

[b19] Hale J. K. Functional Differential Equations (Springer, New York, 1971).

[b20] Xia D.-X. Measure and Integration Theory on Infinite-Dimensional Spaces (Elsevier, Academic Press, Massachusetts, 1972).

[b21] Kolmogorov A. & Fomin S. Elements of the Theory of Functions and Functional Analysis (Dover Publications, New York, 1999).

[b22] Korovkin P. P. Bernstein Polynomials, in Hazewinkel, M., Encyclopedia of Mathematics (Springer, Berlin, 2001).

[b23] Kohonen T. Self-Organization and Associative Memory, vol. 8 (Springer, Berlin, 2012).

[b24] Lin W. & Chen G. Large memory capacity in chaotic artificial neural networks: A view of the anti-integrable limit. IEEE Transactions on Neural Networks 20, 1340–1351 (2009). 19622438 10.1109/TNN.2009.2024148

[b25] Van Den Driessche P. & Zou X. Global attractivity in delayed hopfield neural network models. SIAM Journal on Applied Mathematics 58, 1878–1890 (1998).

[b26] Arenas A., Daz-Guilera A., Kurths J., Moreno Y. & Zhou C. Synchronization in complex networks. Physics Reports 469, 93–153 (2008).

[b27] Schäfer C., Rosenblum M. G., Kurths J. & Abel H.-H. Heartbeat synchronized with ventilation. Nature 392, 239–240 (1998). 9521318 10.1038/32567

[b28] Masoller C. & Marti A. C. Random delays and the synchronization of chaotic maps. Physical Review Letters 94, 134102 (2005). 15903993 10.1103/PhysRevLett.94.134102

[b29] Lin W., Pu Y., Guo Y. & Kurths J. Oscillation suppression and synchronization: Frequencies determine the role of control with time delays. EPL-Europhysics Letters 102, 20003 (2013).

[b30] Murugesan M. S. *Delay Effects on Synchronization in Networks of Dynamical Systems*. Ph.D. thesis, Humboldt-Universität zu Berlin, Mathematisch-Naturwissenschaftliche Fakultät I (2013).

[b31] Pecora L. M. & Carroll T. L. Master stability functions for synchronized coupled systems. Physical Review Letters 80, 2109 (1998).

[b32] Rössler O. E. An equation for continuous chaos. Physics Letters A 57, 397–398 (1976).

[b33] Shrii M. M., Senthilkumar D. & Kurths J. Delay coupling enhances synchronization in complex networks. EPL-Europhysics Letters 98, 10003 (2012).

[b34] Lin W. & Ma H. Synchronization between adaptively coupled systems with discrete and distributed time-delays. IEEE Transactions on Automatic Control 55, 819–830 (2010).

[b35] Ma H. & Lin W. Realization of parameters identification in only locally Lipschitzian dynamical systems with multiple types of time delays. SIAM Journal on Control and Optimization 51, 3692–3721 (2013).

