# Supplementary material for: Basin stability in delayed dynamics
Source: Sci Rep. 2016 Feb 24;6:21449. doi: 10.1038/srep21449 (PMC4764917; doi:10.1038/srep21449)
Supplement: Supplementary Information [file srep21449-s1.pdf]

Supplementary Materials for The Article "Basin stability in delayed dynamics"

Siyang Leng,[1,2] Wei Lin,[1,*] and Jürgen Kurths[2,3,4]

[1]School of Mathematical Sciences, LNSM and Centre for Computational Systems Biology, Fudan University, Shanghai 200433, China

[2]Potsdam Institute for Climate Impact Research (PIK), Potsdam 14473, Germany

[3]Department of Physics, Humboldt University, Berlin 12489, Germany

[4]Institute for Complex Systems and Mathematical Biology, University of Aberdeen, Aberdeen AB24 3UE, United Kingdom

CONTENTS

I. Function space for initial values 2

II. BS in additional benchmark models with time delays 2

A. Time-delayed Van der Pol-Duffing oscillator 2

B. Coupled Stuart-Landau oscillators with time delays 3

III. Stability analysis for selecting the range of coefficients 4

IV. Analytical interval estimation for $S_{\mathfrak{B}}^{(n,\alpha)}$ 5

References 9

* To whom correspondence should be addressed. wlin@fudan.edu.cn

I. FUNCTION SPACE FOR INITIAL VALUES

The Bernstein basis. In addition to the two bases introduced in the main text to construct the function space for the initial values, the Bernstein basis, which is the third basis we use in the main text, can be expressed as

$$B_{j,m}(t) = C_m^j t^j (1-t)^{m-j}, \quad j = 0, 1, \cdots, m, \quad 0 \le t \le 1,$$

where C_m^j is the binomial coefficient [1]. The Bernstein basis, though nonorthogonal, offers valuable insight into its geometrical behavior and can be used to approximate any continuous function f on $[0, 1]$, that is,

$$\sum_{j=0}^{m} f\left(\frac{j}{m}\right) B_{j,m}(x) \to f(x), \text{ as } m \to \infty,$$

where the limit is interpreted in a sense of uniform convergence for continuous functions [2]. For any given n, the Bernstein basis is changing, which is different from the Fourier series where *a priori* given is an orthogonal basis that contains an infinite number of basis functions. Moreover, the coefficients in the above approximation correspond to the values of f on the lattice, so that the Bernstein basis is always used in the curve or surface interpolation. As for our problem, for any given n, we set f_i in $C_{(n,\alpha)}$ as $f_i(x) = B_{i,n}(x)$ and let all coefficients a_i satisfy $\sum_{i=1}^{n} |a_i| < \alpha$ for some given α. Then, akin to the algorithms proposed in the main text, we can compute the nth-order approximate basin stability (BS) for delayed dynamics.

As a matter of fact, some other function set, including a set of stepping (piecewise constant) functions, could be analogously used to define the BS for delayed dynamics. Then, all these functions could be used in the procedure of cross-validation for getting a more steady estimation of the BS.

II. BS IN ADDITIONAL BENCHMARK MODELS WITH TIME DELAYS

We have demonstrated the generalized BS approach with the circumstance of multiple equilibriums in the main text. In fact, it could be applicable to systems with stable periodic orbits and even systems with both kinds of stable steady states, which is illustrated by the following examples.

A. Time-delayed Van der Pol-Duffing oscillator

First, we investigate the time-delayed Van der Pol-Duffing oscillator, where a linear term of delayed position feedback is induced [3]. The equation of motion with x denoting the position of an oscillator is

$$\ddot{x} - (\alpha - \gamma x^2)\dot{x} + \omega_0^2 x + \beta x^3 = A(x_\tau - x), \tag{S1}$$

where α and γ are damping coefficients, β is the rigidity coefficient, and ω_0 is the inherent frequency of the system. In the feedback term, $x_\tau = x(t-\tau)$ with τ denotes the time delay and A is the gain coefficient of feedback. Here, we set $\alpha = 8$ and $\alpha = 15$ for the expansions of the initial values of x and $\dot{x}$, respectively, and denote the BS simply by $S^n_{\mathfrak{B}}$.

Coexistence of multiple periodic orbits and equilibriums of system (S1) can be found when the parameters are properly selected. In particular, we perform our BS computing approach with changing time delay τ in the interval $[4, 8]$. As shown numerically in Fig. S-1, the system has only one periodic orbit when $\tau = 4$, and then with increasing τ, the system bifurcates gradually from two periodic orbits to coexisting states, one equilibrium and one period orbit. Finally, the equilibrium vanishes when τ further increases. Two special cases of $\tau = 5$ and $\tau = 7$ are shown, respectively, in Figs. S-1(a), S-1(d) & S-1(g) and Figs. S-1(b), S-1(e) & S-1(h), where fluctuations of $S^n_{\mathfrak{B}}$ become slight for sufficiently large n and all three bases. Therefore, based on the cross-validation, we use the first $n = 20$ basis functions for the function space of the initial values. Figures S-1(c), S-1(f) & S-1(i) show the variations of $S^{20}_{\mathfrak{B}}$ with increasing τ for the three bases. Figure S-1(j) highlights the normalized curves of $S^{20}_{\mathfrak{B}}$ for the periodic orbit of a larger amplitude for the three bases, which shows a high consensus.

B. Coupled Stuart-Landau oscillators with time delays

The Stuart-Landau oscillator also exhibits co-existence of multiple stable periodic orbits when delayed state feedback terms are induced, which is completely different from the system without delay [4]. We use the generalized BS method to investigate the variation of the basin stability with time delay for the co-existing stable periodic orbits. The Stuart-Landau system with delay feedback terms can be written as:

$$\begin{aligned} \dot{x} &= \alpha x - \omega_0 y - (x^2+y^2)(\alpha x + \beta y) + K[x - x(t-\tau)], \\ \dot{y} &= \omega_0 x + \alpha y - (x^2+y^2)(-\beta x + \alpha y) + K[x - x(t-\tau)], \end{aligned} \tag{S2}$$

where α, β, ω_0 are parameters and K is a gain coefficient. Here, we set $\alpha = 12$ and denote by $S^n_{\mathfrak{B}} = S^{(n,12)}_{\mathfrak{B}}$.

Still shown in Figs. S-2(a), S-2(c) & S-2(e), the fluctuations of $S^n_{\mathfrak{B}}$ become smaller and smaller with increasing n for the three bases. Through observing the variation of $S^n_{\mathfrak{B}}$ in Figs. S-2(b), S-2(d) & S-2(f), we can find that the terminal convergence of system (S2) changes from one stable periodic orbit, to coexistence of two stable periodic orbits, and then to one stable periodic orbit with increasing time delay τ. Similar transition phenomena occur when τ gets further large. Moreover, the normalized variations of $S^{20}_{\mathfrak{B}}$ with τ for the three bases reach a high consensus, as shown in Fig. S-2(g).

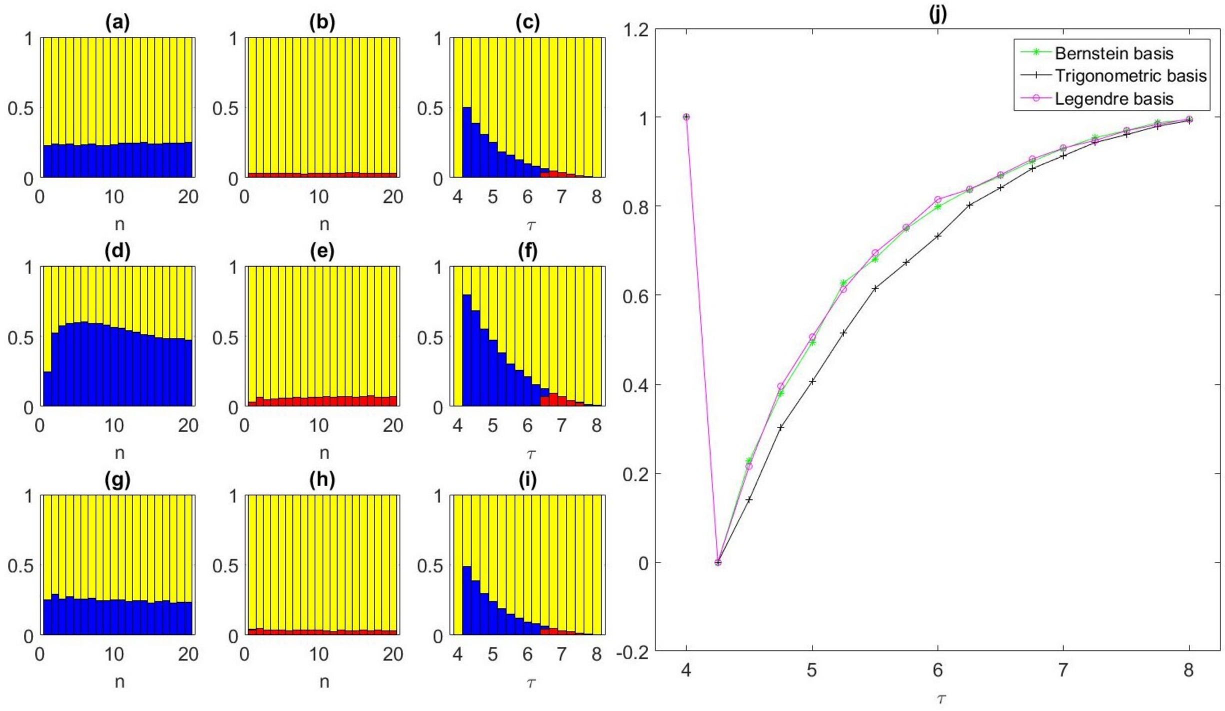

FIG. S-1. **Basin stability of the time-delayed Van der Pol-Duffing oscillator (S1) with respect to three different bases.** In the left nine graphs, red, blue and yellow bars correspond, respectively, to an equilibrium, a periodic orbit with a smaller amplitude, and a periodic orbit with a larger amplitude. Graphs in the first column [(a), (d) and (g) when $\tau = 5$] and in the second one [(b), (e) and (h) when $\tau = 5$] columns depict $S^n_{\mathfrak{B}}$ under three bases (from top to bottom: the Bernstein basis, the trigonometric basis, and the Legendre basis), which display a minor fluctuation of $S^n_{\mathfrak{B}}$ for sufficiently large n. (c), (f) and (i) in the third column show the variations of $S^{20}_{\mathfrak{B}}$ with increasing τ for the three bases. (j) displays the normalized curves of $S^{20}_{\mathfrak{B}}$ for the periodic orbit of a larger amplitude for the three bases, which shows a high consensus.

III. STABILITY ANALYSIS FOR SELECTING THE RANGE OF COEFFICIENTS

In the design of the generalized BS for the delayed dynamics, the n-th order approximate BS, $S^{(n,\alpha)}_{\mathfrak{B}}$, is clearly relevant to the parameter α. Different α may lead to different $S^{(n,\alpha)}_{\mathfrak{B}}$. However, what we are mainly concerned about is the changing tendency $S^{(n,\alpha)}_{\mathfrak{B}}$ with respect to the essential parameters. Thus, it is adequate for us to choose sufficiently large α for calculating the BS. Moreover, for each coefficient a_i in front of the basis functions, we still need to determine the range $[\gamma_1, \gamma_2]$ from which we can sample the values. Once all the sampled a_i satisfy $\sum_{i=1}^{n} a_i^2 \leq \alpha^2$, the corresponding function is taken as an initial value for the delayed dynamics.

Here, we use the time-delayed Van der Pol-Duffing oscillator model (S1) to show the stability of $S^{(n,\alpha)}_{\mathfrak{B}}$

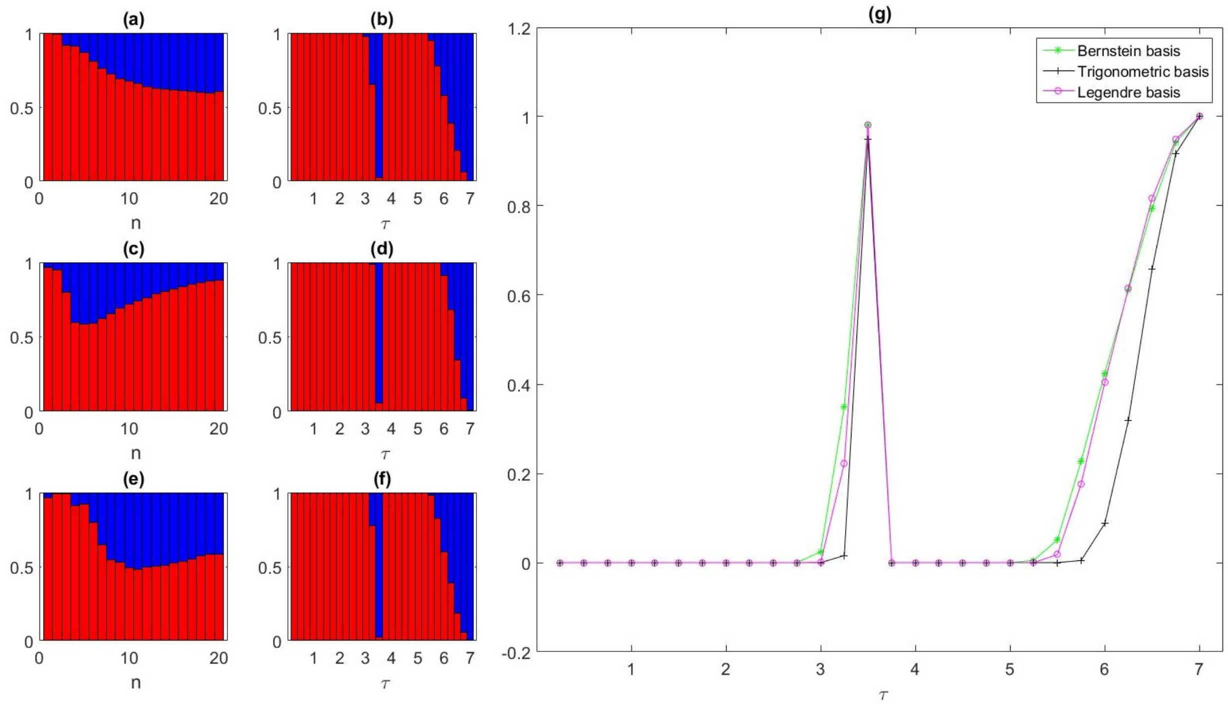

FIG. S-2. **Basin stability of the delay-coupled Stuart-Landau system (S2) with respect to three different bases.** In the left six plots, red and blue bars correspond to stable periodic orbits with a smaller amplitude and a larger amplitude, respectively. (a), (c) and (e) show the variations of $S_{\mathfrak{B}}^{n}$ with n for the three bases (from top to bottom: the Bernstein basis, the trigonometric basis, and the Legendre basis). (b), (d) and (f) show the variation of the generalized BS, $S_{\mathfrak{B}}^{20}$, with τ. (g) displays the normalized curves of $S_{\mathfrak{B}}^{20}$ for the stable periodic orbit with a larger amplitude, which shows a consensus for the three bases.

with respect to the range for each coefficient a_i. For simplicity, in this example, we choose the coefficients from the range $[-\gamma, \gamma]$ for the initial value of x, and take the coefficients from $[-1.875\gamma, 1.875\gamma]$ for the initial value of $\dot{x}$. We investigate the variation of $S_{\mathfrak{B}}^{(20,\alpha)}$ with γ, where α's values are the same as those used in Sec. II A. As shown in Fig. S-3, when γ becomes larger, $S_{\mathfrak{B}}^{(20,\alpha)}$ tends to be quite stable for all the bases we use. Therefore, for a given α, taking a sufficiently large γ results in a much accurate computation of the generalized BS.

IV. ANALYTICAL INTERVAL ESTIMATION FOR $S_{\mathfrak{B}}^{(n,\alpha)}$

Here, we analytically establish an interval estimation for $S_{\mathfrak{B}}^{(n,\alpha)}$ while n is sufficiently large. It is impossible to perform a random sampling of the initial values directly on the function space E_α which could be

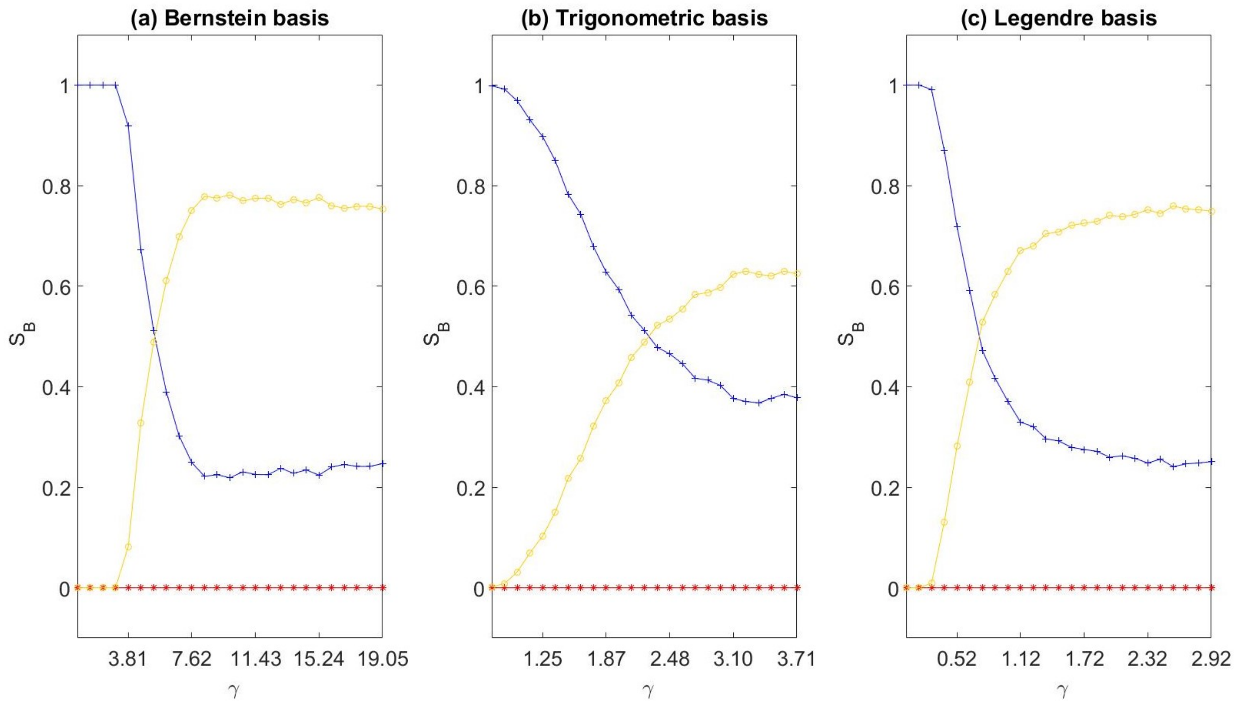

FIG. S-3. **Stability analysis for computing the BS versus the range width of the coefficients.** For the three bases and given α, when the boundary parameter γ for the coefficients becomes larger, $S_{\mathfrak{B}}^{(20,\alpha)}$ tends to be relatively stable. Here, the same as the color settings in Fig. S-1, the curves' colors correspond to the three steady states in the time-delayed Van der Pol-Duffing oscillator (S1).

written as:

$$E_\alpha = \left\{ g = a_1 f_1 + a_2 f_2 + \cdots + a_n f_n + \cdots \;\middle|\; \sum_{i=1}^{\infty} a_i^2 < \alpha^2 \right\},$$

where $\{f_1, f_2, \cdots, f_n, \cdots\} \subset C[-\tau, 0]$ is a given standard orthogonal basis. We thus consider a series of simplified function spaces:

$$E_{(n,\alpha)} = \left\{ g_n = a_1 f_1 + a_2 f_2 + \cdots + a_n f_n \;\middle|\; a_i \in \left\{0, \pm\frac{1}{2^n}\gamma, \pm\frac{2}{2^n}\gamma, \cdots, \pm\gamma\right\},\; \sum_{i=1}^{n} a_i^2 \le \alpha^2 \right\},\;\; n = 1, 2, \cdots.$$

Obviously, we have $E_{(1,\alpha)} \subset E_{(2,\alpha)} \subset \cdots \subset E_{(n,\alpha)} \subset \cdots$ and $\lim_{n\to\infty} E_{(n,\alpha)} = E_\alpha$. The following sampling analysis is then based on these finite sets $E_{(n,\alpha)}$ with their cardinalities $N \triangleq |E_{(n,\alpha)}| \le (2^n + 1)^n$.

Consider a stable steady state, denoted by $\mathcal{A}$. Then the corresponding sampling population becomes $U = \{Z_1, Z_2, \cdots, Z_N\}$, where $Z_i = 1$ represents the i-th initial value in $E_{(n,\alpha)}$ from which the trajectory of the delayed dynamics is attracted to $\mathcal{A}$, and otherwise $Z_i = 0$. We sample k functions from U and define an indicator function as follows:

$$D_i = \begin{cases} 1, & \text{when } Z_i \text{ is sampled,} \\ 0, & \text{when } Z_i \text{ is not sampled.} \end{cases}$$

We intend to estimate the proportion of $N_1 = \sum_{i=1}^{N} Z_i$ in N, i.e., $\overline{Z} = \frac{N_1}{N} = p$, which is equal to $S_{\mathfrak{B}}^{(n,\alpha)}$. To this end, we need to use the following well-known Wald-Wolfowitz Theorem [5].

Wald-Wolfowitz Theorem. *Suppose that* $\{a_{N1}, \cdots, a_{NN}\}$ *and* $\{x_{N1}, \cdots, x_{NN}\}$ *are two sequences of real numbers. Also suppose that these sequences satisfy*

$$\frac{\frac{1}{N}\sum_{i=1}^{N}(a_{Ni} - \overline{a}_N)^r}{\left[\frac{1}{N}\sum_{i=1}^{N}(a_{Ni} - \overline{a}_N)^2\right]^{\frac{r}{2}}} = O(1), \quad \frac{\frac{1}{N}\sum_{i=1}^{N}(x_{Ni} - \overline{x}_N)^r}{\left[\frac{1}{N}\sum_{i=1}^{N}(x_{Ni} - \overline{x}_N)^2\right]^{\frac{r}{2}}} = O(1),$$

for $r = 3, 4$ *and larger* N*, where*

$$\overline{a}_N = \frac{1}{N}\sum_{i=1}^{N} a_{Ni}, \quad \overline{x}_N = \frac{1}{N}\sum_{i=1}^{N} x_{Ni}.$$

For each N*,* $(X_1, \cdots, X_N)$ *is supposed to be a random vector uniformly taking value from all the permutations of the sequence* $\{x_{N1}, \cdots, x_{NN}\}$ *and denote by*

$$L_N = \sum_{i=1}^{N} a_{Ni} X_i.$$

Then, we have

$$\mathbb{E}(L_N) = N\overline{a}_N\overline{x}_N, \quad \mathrm{Var}(L_N) = \frac{1}{N-1}\left[\sum_{i=1}^{N}(a_{Ni} - \overline{a}_N)^2\right]\left[\sum_{i=1}^{N}(x_{Ni} - \overline{x}_N)^2\right].$$

Moreover, the probability for L_N *has the following convergence law:*

$$\mathbb{P}\left\{\frac{L_N - \mathbb{E}(L_N)}{\sqrt{\mathrm{Var}(L_N)}} \leq z\right\} \to \frac{1}{\sqrt{2\pi}}\int_{-\infty}^{z} \mathrm{e}^{-\frac{1}{2}t^2}\mathrm{d}t,$$

as $N \to \infty$.

In order to use the theorem above for solving our problem, we choose

$$\{a_{N1}, \cdots, a_{NN}\} = \{Z_1, \cdots, Z_N\}, \quad \{x_{N1}, \cdots, x_{NN}\} = \left\{\frac{1}{k}, \cdots, \frac{1}{k}, 0, \cdots, 0\right\},$$

so that $y \triangleq L_N = \frac{1}{k}\sum_{i=1}^{N} Z_i D_i$. It is easy to check that these settings satisfy the conditions required in the theorem above. In what follows, we compute $\mathbb{E}(L_N)$ and $\mathrm{Var}(L_N)$. On the one hand,

$$\mathbb{E}(L_N) = \mathbb{E}\left(\frac{1}{k}\sum_{i=1}^{N} Z_i D_i\right) = \frac{1}{k}\sum_{i=1}^{N} Z_i \mathbb{E}(D_i) = \overline{Z} = p.$$

On the other hand,

$$
\begin{aligned}
\mathrm{Var}(L_N) &= \mathrm{Var}\left(\frac{1}{k}\sum_{i=1}^{N} Z_i D_i\right) \\
&= \frac{1}{k^2}\left[\sum_{i=1}^{N} Z_i^2 \mathrm{Var}(D_i) + \sum_{i,j,i\neq j}^{N} Z_i Z_j \mathrm{cov}(D_i, D_j)\right] \\
&= \frac{1}{k^2}\left[\frac{k}{N}\left(1-\frac{k}{N}\right)\sum_{i=1}^{N} Z_i^2 - \frac{k}{N(N-1)}\left(1-\frac{k}{N}\right)\sum_{i\neq j}^{N} Z_i Z_j\right] \\
&= \frac{1}{k}\left(1-\frac{k}{N}\right)\frac{1}{N-1}\left[\sum_{i=1}^{N} Z_i^2 - \frac{1}{N}\left(\sum_{i=1}^{N} Z_i\right)^2\right] \\
&= \frac{N-k}{k(N-1)} p(1-p),
\end{aligned}
$$

where the last equality is due to $\sum_{i=1}^{N} Z_i^2 = \sum_{i=1}^{N} Z_i = N_1$. By the Wald-Wolfowitz Theorem, we have

$$
u = \frac{y-p}{\sqrt{\frac{N-k}{k(N-1)} p(1-p)}} \sim \mathrm{N}(0,1),
$$

as $N \to \infty$. Here, $\mathrm{N}(0,1)$ represents the standard normal distribution. Given a confidence level $1-\beta$, we have an interval estimation for p as follows:

$$
\begin{aligned}
1-\beta &\approx \mathbb{P}\left\{\left|\frac{y-p}{\sqrt{\frac{N-k}{k(N-1)} p(1-p)}}\right| \le u_{1-\frac{\beta}{2}}\right\} \\
&= \mathbb{P}\left\{y - u_{1-\frac{\beta}{2}}\sqrt{\frac{N-k}{k(N-1)} p(1-p)} \le p \le y + u_{1-\frac{\beta}{2}}\sqrt{\frac{N-k}{k(N-1)} p(1-p)}\right\}, \qquad \text{(S3)}
\end{aligned}
$$

where $u_{1-\frac{\beta}{2}}$ is the $\left(1-\frac{\beta}{2}\right)$-quantile of the distribution $\mathrm{N}(0,1)$. Solving the inequality for p in (S3) yields:

$$
P(P_L \le p \le P_U) \approx 1-\beta
$$

as N is sufficiently large, where

$$
P_{L,U} = \frac{\left[2y + \frac{u^2_{1-\frac{\beta}{2}}(N-k)}{k(N-1)}\right] \mp \sqrt{4y(1-y)\frac{u^2_{1-\frac{\beta}{2}}(N-k)}{k(N-1)} + \left[\frac{u^2_{1-\frac{\beta}{2}}(N-k)}{k(N-1)}\right]^2}}{2\left(1 + \frac{u^2_{1-\frac{\beta}{2}}(N-k)}{k(N-1)}\right)}.
$$

The above argument manifests that for a sufficiently large N, the estimated interval $[P_L, P_U]$ for $p = S_{\mathfrak{B}}^{(n,\alpha)}$ with an increase of k, reduces to a fairly small interval in which p is located with a large probability.

As an illustrative example, we still use the time-delayed Van der Pol-Duffing oscillator (S1) with $\tau = 5$. We adopt the Bernstein basis to estimate the interval of $[P_L, P_U]$ within a confidence level $1-\beta =$

99%. Accordingly, $u_{1-\frac{\beta}{2}} = 2.576$. As shown in Fig. S-4, for both cases of $n = 5$ and $n = 10$, $[P_L, P_U]$ reduces gradually to a very small interval with an increase of k, which confirms the analytical argument performed above. More importantly, it confirms the reliability of the algorithm introduced in the main text for computing the n-th order approximate BS.

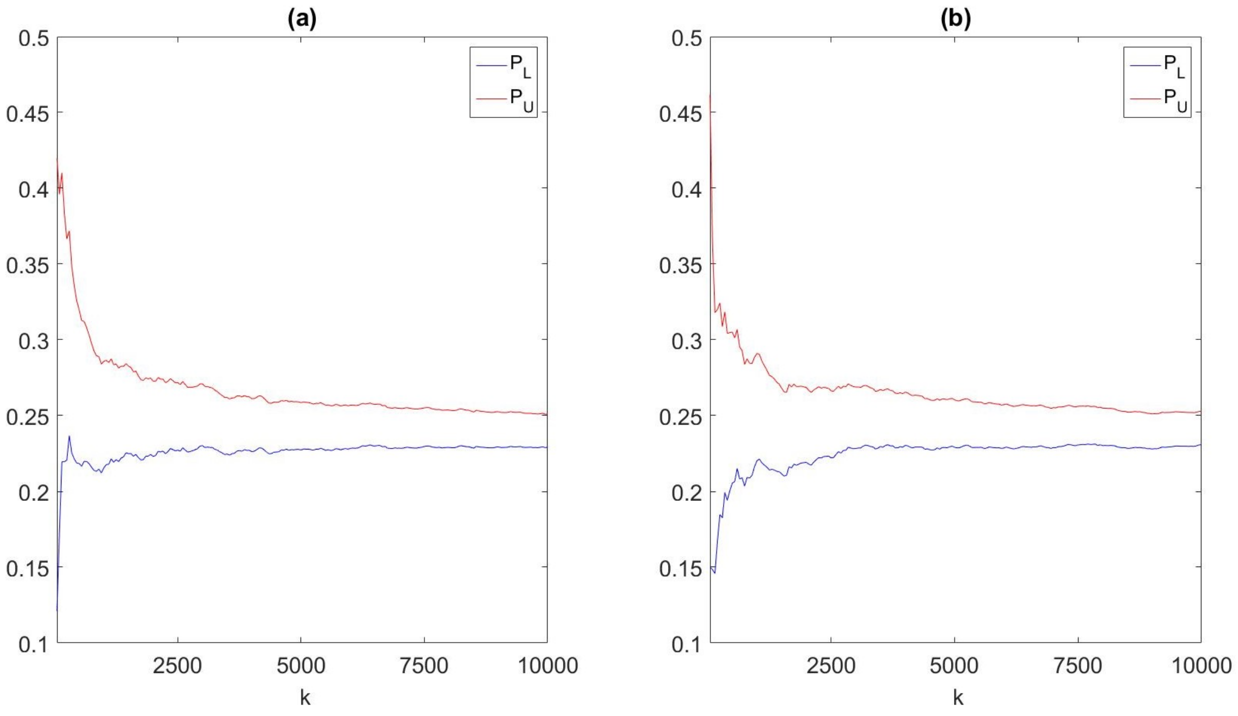

FIG. S-4. **An interval estimation for $S_{\mathfrak{B}}^{(n,\alpha)}$ in the time-delayed Van der Pol-Duffing oscillator (S1).** Here, we take $n = 5$ (a) and $n = 10$ (b).

[1] R. T. Farouki. Legendre–bernstein basis transformations. *Journal of Computational and Applied Mathematics*, 119(1-2):145–160, July 2000.

[2] P. J. Davis. *Interpolation and Approximation*. Dover, New York, 1975.

[3] J. Xu and K. W. Chung. Effects of time delayed position feedback on a van der pol-duffing oscillator. *Physica D-Nonlinear Phenomena*, 180(1-2):17–39, 2003.

[4] H. Shang. Multiple periodic solutions in linear delayed state feedback controlled stuart-landau system. *Journal of Shanghai Institute of Technology*, 2010(1):22–25, 2010.

[5] A. Wald and J. Wolfowitz. Statistical tests based on permutations of the observations. *Annals of Mathematical Statistics*, 15(1):358–372, 1944.
